# Diagnosis and Management of Fetal and Neonatal Thyrotoxicosis

**DOI:** 10.3390/medicina59010036

**Published:** 2022-12-23

**Authors:** Roxana-Elena Bohîlțea, Bianca-Margareta Mihai, Elena Szini, Ileana-Alina Șucaliuc, Corin Badiu

**Affiliations:** 1 Department of Obstetrics and Gynecology, ‘Carol Davila’ University of Medicine and Pharmacy, 020021 Bucharest, Romania; 2 Department of Obstetrics and Gynecology, Filantropia Clinical Hospital, 011132 Bucharest, Romania; 3 Doctoral School, ‘Carol Davila’ University of Medicine and Pharmacy, 020021 Bucharest, Romania; 4 Department of Neonatology, University Emergency Hospital, 050098 Bucharest, Romania; 5 Department of Thyroid Disorders, ‘C.I. Parhon’ National Institute of Endocrinology, 011863 Bucharest, Romania; 6 Department of Endocrinology, ‘Carol Davila’ University of Medicine and Pharmacy, 020021 Bucharest, Romania

**Keywords:** fetal thyrotoxicosis, Graves’ disease, TSH receptor stimulating antibodies, fetal tachycardia

## Abstract

*Background and Objectives*: Clinical fetal thyrotoxicosis is a rare disorder occurring in 1–5% of pregnancies with Graves’ disease. Although transplacental passage of maternal TSH receptor stimulating autoantibodies (TRAb) to the fetus occurs early in gestation, their concentration in the fetus is reduced until the late second trimester, and reaches maternal levels in the last period of pregnancy. The mortality of fetal thyrotoxicosis is 12–20%, mainly due to heart failure. *Case report*: We present a case of fetal and neonatal thyrotoxicosis with favorable evolution under proper treatment in a 37-year-old woman. From her surgical history, we noted a thyroidectomy performed 12 years ago for Graves’ disease with orbitopathy and ophthalmopathy; the patient was hormonally balanced under substitution treatment for post-surgical hypothyroidism and hypoparathyroidism. From her obstetrical history, we remarked a untreated pregnancy complicated with fetal anasarca, premature birth, and neonatal death. The current pregnancy began with maternal euthyroid status and persistently increased TRAb, the value of which reached 101 IU/L at 20 weeks gestational age and decreased rapidly within 1 month to 7.5 IU/L, probably due to the placental passage, and occurred simultaneously with the development of fetal tachycardia, without any other fetal thyrotoxicosis signs. In order to treat fetal thyrotoxicosis, the patient was administered methimazole, in addition to her routine substitution of 137.5 ug L-Thyroxine daily, with good control of thyroid function in both mother and fetus. *Conclusions*: Monitoring for fetal thyrotoxicosis signs and maternal TRAb concentration may successfully guide the course of a pregnancy associated with Graves’ disease. An experienced team should be involved in the management.

## 1. Introduction

Fetal thyrotoxicosis represents a rare disorder that complicates 1 out of 4000–50,000 pregnancies. The most important etiology of fetal thyrotoxicosis is represented by Graves’ disease [1]. Other rare involved etiologies are thyrotropin-receptor activating mutations [2,3] and an alpha subunit G protein-activating mutation, known as McCune–Albright syndrome [4]. In pregnant patients suffering from Graves’ disease, 1 out of 70 pregnancies will develop fetal thyrotoxicosis, while pregnant patients requiring antithyroid treatment in the last trimester have a 22% chance of delivering a baby with neonatal thyrotoxicosis [5]. Thyroid stimulating hormone (TSH) is synthesized beginning at the 10th–12th gestational week, when the fetal thyroid begins to concentrate iodine and has the ability to produce iodothyronines. The hormone production is quantitatively reduced until the 18th–20th week of gestation, after which it intensifies gradually [6]. In the first trimester, the maternal thyroid hormones are fundamental for the normal development of the fetus in the absence of a functional fetal thyroid. The placental passage of maternal thyroid hormones has been demonstrated in newborns with congenital absence of the thyroid, where cord serum concentrations of thyroid hormones were between 20 and 50% of those of euthyroid newborns [7]. Fetal, followed by neonatal, thyrotoxicosis occurs when transplacental passage of the TSH receptor antibodies (TRAb) reaches a high serum concentration, and it is increased in the second half of the pregnancy, when the placental permeability reaches a maximum capacity [8]. TRAb could either stimulate the fetal thyroid, resulting in thyrotoxicosis, or have an inhibiting effect, inducing fetal hypothyroidism [9]. Fetal thyrotoxicosis is considered to be a rare disease due to the fact that maternal immunity in pregnancy is modulated, and, therefore, the TRAb serum concentration should diminish [10]. Maternal medical history of Graves’ disease, despite being euthyroid under anti-thyroid therapy or in substitution treatment after radioiodine treatment or thyroidectomy, is of the utmost importance considering the potentially elevated TRAb serum levels [11]. Studies have shown that fetal and neonatal thyrotoxicosis occurs only in women with three to five times the normal serum concentrations of stimulating TRAb [9]. Regarding the neonatal prognosis, the majority of newborns from mothers suffering from Graves’ disease has goiter. Newborns with manifest thyrotoxicosis are recognized by restlessness, jitteriness, irritability, tachycardia, arrhythmias, systemic and pulmonary hypertension, cardiac failure, periorbital edema, exophthalmia, lid retraction, insatiable appetite, diarrhea, weight loss, sweating, flushing, hepatosplenomegaly, thymic enlargement, persisting acrocyanosis, lymphadenopathy, advanced bone age, microcephaly or craniosynostosis [12]. Neonatal improvement is strongly associated with the moment of maternal TRAb disappearance from fetal circulation [12]. The fetal prognosis is mostly favorable with the optimum therapy promptly instituted. Unfortunately, however, some infants have low intelligence quotients despite being treated for neonatal thyrotoxicosis, suggesting the complexity of fetal or neonatal hyperthyroidism’s effect on the developing central nervous system. Other long-term complications of neonatal thyrotoxicosis are craniosynostosis, growth retardment, hyperactivity and behavioral issues [13]. Rarely, neonatal Graves’ disease may convert into central hypothyroidism with decreased TSH secretion, due to intrauterine exposure to elevated serum thyroid hormone concentrations during a vital phase of development [14]. 

We present the case of fetal, followed by neonatal, thyrotoxicosis with good neonatal evolution under substitution therapy combined with thioamides in a pregnant woman with prior thyroidectomy, with elevated TRAb serum concentration from the beginning of the pregnancy. 

## 2. Case Presentation

A 37-year-old pregnant Caucasian female, 170 cm and 65 kg, presented in our medical unit for pregnancy monitoring. She was known to have Graves’ disease and orbitopathy, being euthyroid in substitution treatment with Levothyroxine 112.5 μg, 1 g calcium carbonate in association with 1 μg alfacalcidol daily, for postoperative hypothyroidism and hypoparathyroidism after a thyroidectomy performed 12 years prior. From the patient’s obstetrical history, we noted a miscarriage and an iatrogenic premature delivery, which occurred at the age of 29 by emergency Cesarean section at 31 gestational weeks, of a 1400 g newborn with cardiac insufficiency due to manifest fetal hyperthyroidism with goiter, tachycardia, hydrops and associating fetal cerebral ventriculomegaly, gradually progressing to postpartum hydrocephaly. The newborn deceased at 21 days postpartum due to bronchopulmonary complications. The evolution of this previous pregnancy was marked by a massive increase of the TRAb serum concentration, with no treatment for the fetal hyperthyroidism. 

The actual pregnancy started under neural tube defect protection with 400 μg folic acid and 400 μg calcium L-methylfolate, 400 IU vitamin E, enoxaparin 4000 IU and 150 mg aspirin daily for the thromboembolic disease prophylaxis in the context of the existing 1691G>A heterozygote Factor V Leiden mutation. The levothyroxine dose was increased to 150 μg daily. A non-invasive prenatal DNA screening test was performed for the entire genome at 12 gestational weeks. A fetal fraction of 7.83% was tested and it revealed low risk for the trisomies 21, 18, 13, 9, 16 and 22; aneuploidies XO, XXX, XXY and XYY; and for the microdeletion or duplication syndromes. 

**Figure 1:**
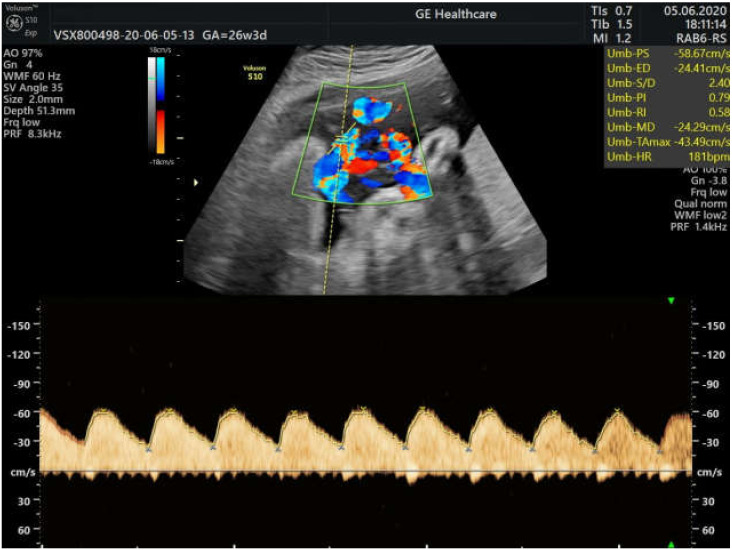
Fetal tachycardia at 26 weeks of gestation despite reducing the daily Levothyroxine dose.

The normal course of the pregnancy was disturbed at 23 gestational weeks, when the fetal heart rate began to increase up to 180 bpm and we observed a fetal hyperdynamic status. At that moment, there were no fetal signs of cardiac failure or goiter. One week later, the levothyroxine dose was decreased to 137.5 μg daily. As there was no improvement in the fetal heart rate (Figure 1), in collaboration with the endocrinologist, a dose of 10 mg methimazole daily was introduced into the patient’s treatment in the 26th gestational week. 

**Figure 2:**
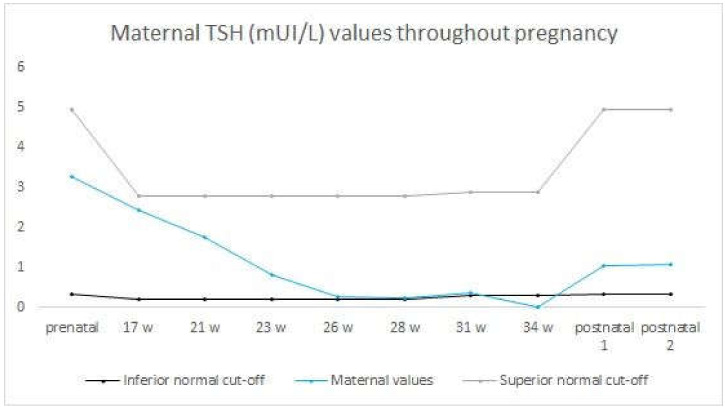
Maternal TSH values before pregnancy, throughout pregnancy and postnatally as compared to normal TSH values in pregnant and non-pregnant women The maternal TSH values were monitored before and during the pregnancy, with a monthly frequency at the beginning of the second trimester; further, with the appearance of the ultrasonographic signs of fetal thyrotoxicosis, the monitoring frequency increased. The TSH values had a descending tendency with the advancing pregnancy.

**Figure 3:**
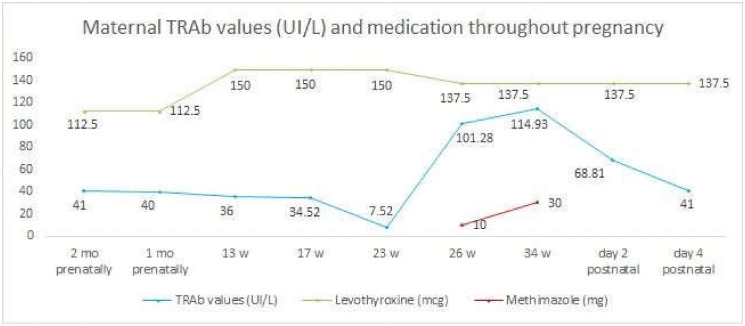
Maternal TRAb levels before pregnancy, throughout pregnancy and postnatally. The decrease of TRAb values in the 23rd gestational week corresponded to the appearance of fetal thyrotoxicosis ultrasonographic signs. The Levothyroxine was increased in the first trimester, with a decrease in the 23rd gestational week. Methimazole was introduced in the 26th gestational week.

During the 30th gestational week, the patient was administered four doses of 8 mg dexamethasone to aid in the acceleration of fetal lung maturity. The pregnancy was closely monitored using ultrasound markers. We examined the early signs of fetal cardiac failure, the size and vascularization of the fetal thyroid, the fetal heart rate, the maternal TRAb serum concentration and the thyroid function tests (Figure 2). The methimazole dose was gradually increased, as our patient, at 31st gestational weeks, received 30 mg methimazole daily, divided into three doses (Figure 3). 

**Figure 4:**
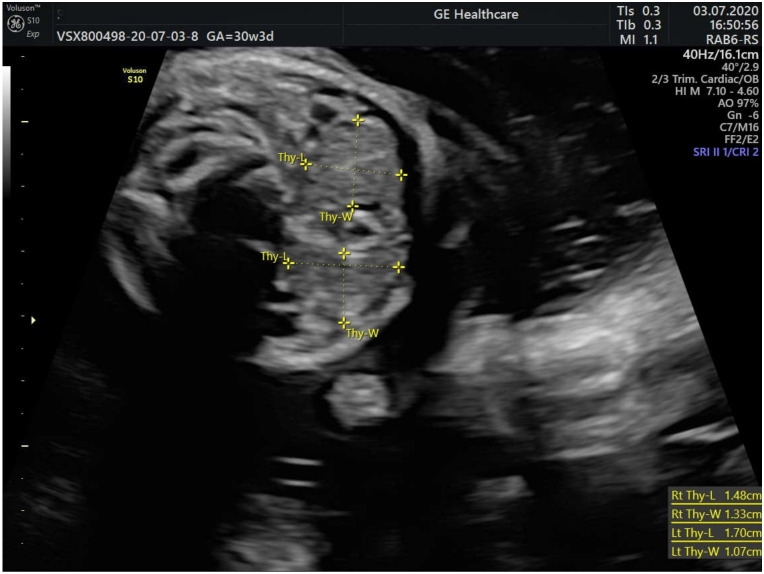
2-D Ultrasonographic measurements of the fetal thyroid at 30 weeks and 3 days of gestation: right thyroid lobe measures 1.48/1.33 cm and the left thyroid lobe measures 1.70/1.07 cm.

We monitored the fetal thyroid vascularization and dimensions, which were in the normal range according to the gestational age (Figure 4). 

Due to fetal status deterioration, which was suspected at the ultrasound evaluation of the Doppler parameters and the fetal biophysical profile, an emergency Cesarean section was performed at 35 weeks of gestation, and a male fetus weighing 2530 g and 50 cm in length was delivered, with an APGAR score of 6 at 1 min and 8 at 5 min. Mild perinatal hypoxia, a tight double nuchal cord and the presence of meconium in the amniotic fluid were noticed. The patient received ablactation treatment with cabergoline to decrease the risk of further TRAb passage to the fetus. The newborn had a 32 cm fronto-occipital head circumference, a 30 cm chest circumference, mild exophthalmia, moderate congenital ventriculomegaly (occipital horns of the lateral ventricles of 14 mm and, respectively, 16 mm), no clinical or ultrasound heart abnormalities and normal heart rate. The fetal thyroid function tests were performed at birth from the cord blood, confirming the fetal thyrotoxicosis with fT4 > 100 pmol/L, T3 = 8.4 nmol/L (N = 1.23–4.22 nmol/L) and TRAb 39.74 UI/L, and were also performed at 48 h and 72 h after delivery. After that, the newborn was closely monitored and administered thiamazole at 0.5–2 mg/kg bodyweight daily, beginning with the second day of life, with dose adjustment depending on the thyroid hormone values and with liver function under close monitoring. Propranolol was also administered at a dose of 2 mg/kg bodyweight daily in order to control the fetal tachycardia. The medication was decreased gradually as the newborn’s clinical status improved and the TRAb serum concentration declined. At 2 months, the baby had a significant decrease in TRAb serum concentration (2.45 UI/L) with subclinical hyperthyroidism (TSH < 0.005, fT4 9.8 pmol/L), and presented a normal development. 

## 3. Discussion

The particularity of this case consists of a pregnant patient who had very high TRAb serum levels from the beginning of the pregnancy, after 12 years since the thyroidectomy, with the initiation of the thioamide treatment in the second trimester with good fetal and neonatal prognosis.

The 2017 American Thyroid Association (ATA) Guidelines for the Diagnosis and Management of Thyroid Disease during Pregnancy [15] and the Postpartum, as well as the 2018 European Thyroid Association (ETA) Guideline for the Management of Graves’ Hyperthyroidism [16], are against iodine ablative therapy for Graves’ disease treatment during pregnancy. A future pregnancy should be postponed for 6 months until the patient is euthyroid, after iodine ablative therapy and after introducing levothyroxine substitution treatment. In addition, ETA does not recommend iodine ablative therapy while breastfeeding [16]. ATA recommends surgical treatment in cases of thyrotoxicosis resistant to high doses of thioamides, and the timing of the intervention should be in the second trimester [17]. ETA recommends thyroidectomy in cases of allergy or contraindications to thioamides [16]. Besides the risk of infection or bleeding, the risk of parathyroid glands injuries is not negligible, so it is fundamental to evaluate the need for active vitamin D supplementing in order to avoid additional fetal and neonatal adverse outcomes [18].

Regarding thioamide drugs (propylthiouracil, methimazole and carbimazole), both propylthiouracil (PTU) and methimazole (MMI) are associated with the risk of birth defects: MMI has a risk of 3–4% [19] and PTU 2–3%, but less severe [20]. ATA and ETA recommend avoiding antithyroid drugs in the first trimester, but if the case imposes immediate treatment, PTU is preferred for this gestational period [16,21]. After 16 gestational weeks, ETA recommends switching PTU to MMI if medical treatment is required, as well as measuring the maternal FT4 and TSH every two weeks after treatment initiation [16]. Thioamides inhibit the production of thyroid hormones. The main side effects occurring in the mother are allergic reactions, agranulocytosis (0.15%) and liver failure (<0.1%) [22,23]. When recommending PTU therapy, it is advisable to monitor hepatic enzymes for the risk of liver failure. Besides having teratogenic effects, thioamides have been associated with certain birth defects. MMI carries the risk of aplasia cutis, dysmorphic facies, choanal or esophageal atresia, abdominal wall defects (omphalocele), ventricular sept defects and eye and urinary system abnormalities [19,24,25]. PTU is linked with fetal face and neck cysts, as well as with urinary tract anomalies occurring in males [15]. In our case, we delayed the thioamide treatment and introduced thiamazole in the 26th gestational week, after excluding a possible iatrogenic thyrotoxic effect of levothyroxine on the fetus and, thus, avoiding the risk of fetal malformations due to thioamides in the first trimester. 

In the literature, the possibility of performing a scintigraphy to assess the remnant tissue of the thyroid gland after thyroidectomy, or an ectopic localization of thyroid tissue in order to evaluate the best candidates for radioiodine ablation therapy for Graves’ orbitopathy, have been described [26,27]. In our case, the patient did not benefit from this type of investigation prior to the pregnancy.

An article published by Batra et al. in 2015 [28] describes the difficulties encountered while managing two cases of severe thyrotoxicosis during pregnancy, with fetal and subsequent neonatal thyrotoxicosis in a woman with subtotal thyroidectomy for recurrent thyrotoxicosis, followed by hypothyroidism. the authors concluded that early diagnosis and proper treatment may improve fetal morbidity and mortality. Moreover, another case report, published by Sato et al. in 2014 [29], communicates the positive effect of thioamides, more specifically of methimazole, when administered to a pregnant woman who received radical treatment 7 years prior to pregnancy and who presented with oligohydramnios, fetal tachycardia, fetal goiter and accelerated bone maturation in the early third trimester of pregnancy. After 2 weeks of treatment, the fetal heart rate and amniotic fluid index were normal for the gestational age, and after 4 weeks, the fetal thyroid had a normal circumference.

ATA and ETA recommend testing TRAb serum concentration in early pregnancy, along with a laboratory assessment of the TF in patients presenting a history of Graves’ disease treated with ablation, either surgically or with radioiodine. In case of a high maternal TRAb serum concentration, the testing should be repeated between 18–22 gestational weeks [16,30]. If a high TRAb serum concentration is detected (more than three times the upper normal maternal limit or more than 5 IU/L), according to ATA, the mother is counseled to receive thioamides in the third trimester, and further TRAb testing should be conducted between 30–34 gestational weeks in order to estimate the need for the newborn to be monitored and, if necessary, to receive treatment [30]. Besides monitoring the maternal hormones, our monthly maternal TRAb serum concentration monitoring plan was extremely helpful in observing the dynamics of the antibodies and in identifying the moment of the placental TRAb passage to the fetus. This corresponded with the appearance of the early fetal thyrotoxicosis signs, namely fetal tachycardia and fetal hyperdynamic status. 

Another particularity is represented by the fact that, even though most newborns from mothers with Graves’ disease present a goiter, in our case, despite the presence of the manifest neonatal hyperthyroidism, the newborn did not have a goiter.

**Figure 5:**
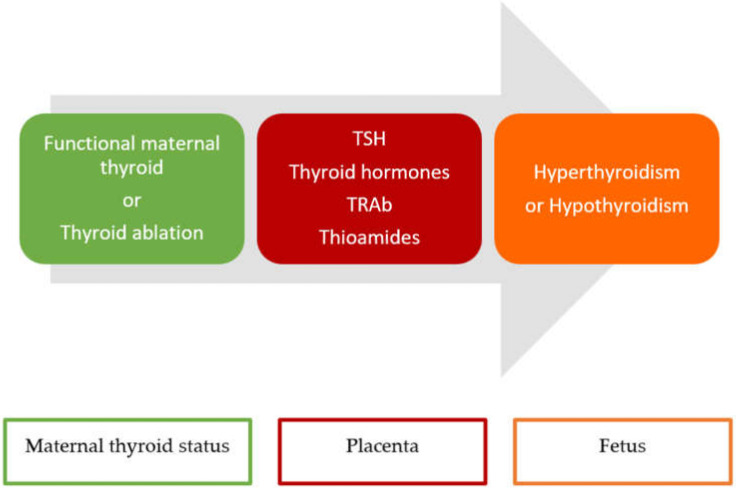
Transplacental passage of maternal hormones, medication and antibodies during pregnancy in women with or without thyroidectomy; TSH = thyroid stimulating hormone; TRAb = TSH receptor antibodies.

Finally, Graves’ disease being an autoimmune disease, despite the fact that our patient received radical treatment, the autoimmune disease remained active and could endanger each and every future pregnancy. Thus, it is important to monitor the maternal TRAb and the hormonal status of the mother, considering the transplacental passage that is currently incompletely discovered and explained in the current literature, and which occurs in every pregnancy (Figure 5).

## 4. Conclusions

The first signs of fetal thyrotoxicosis are fetal tachycardia and fetal hyperdynamic status. We consider it fundamental to monitor the maternal TRAb and thyroid function monthly in order to detect the passage of transplacental antibodies to the fetus, and to establish the optimum treatment so that the mother and fetus have the chance for a normal life. A substitution therapy combined with thioamides could be recommended in cases of pregnant women with postprocedural hypothyroidism and active autoimmune disease.

## Data Availability

The datasets used and/or analyzed during the current study are available from the corresponding author upon reasonable request.
